# Meta-analysis of structural and functional alterations of brain in patients with attention-deficit/hyperactivity disorder

**DOI:** 10.3389/fpsyt.2022.1070142

**Published:** 2023-01-06

**Authors:** Miaomiao Yu, Xinyu Gao, Xiaoyu Niu, Mengzhe Zhang, Zhengui Yang, Shaoqiang Han, Jingliang Cheng, Yong Zhang

**Affiliations:** ^1^Department of Magnetic Resonance Imaging, The First Affiliated Hospital of Zhengzhou University, Zhengzhou, China; ^2^Key Laboratory for Functional Magnetic Resonance Imaging and Molecular Imaging of Henan Province, Zhengzhou, China; ^3^Engineering Technology Research Center for Detection and Application of Brain Function of Henan Province, Zhengzhou, China; ^4^Engineering Research Center of Medical Imaging Intelligent Diagnosis and Treatment of Henan Province, Zhengzhou, China; ^5^Key Laboratory of Magnetic Resonance and Brain Function of Henan Province, Zhengzhou, China; ^6^Key Laboratory of Brain Function and Cognitive Magnetic Resonance Imaging of Zhengzhou, Zhengzhou, China; ^7^Key Laboratory of Imaging Intelligence Research Medicine of Henan Province, Zhengzhou, China

**Keywords:** meta-analysis, ADHD, voxel-based morphometry, functional MRI, neuroimaging

## Abstract

**Background:**

A large and growing body of neuroimaging research has concentrated on patients with attention-deficit/hyperactivity disorder (ADHD), but with inconsistent conclusions. This article was intended to investigate the common and certain neural alterations in the structure and function of the brain in patients with ADHD and further explore the differences in brain alterations between adults and children with ADHD.

**Methods:**

We conducted an extensive literature search of whole-brain voxel-based morphometry (VBM) and functional magnetic resonance imaging (fMRI) studies associated with ADHD. Two separate meta-analyses with the seed-based d mapping software package for functional neural activation and gray matter volume (GMV) were carried out, followed by a joint analysis and a subgroup analysis.

**Results:**

This analysis included 29 VBM studies and 36 fMRI studies. Structurally, VBM analysis showed that the largest GMV diminutions in patients with ADHD were in several frontal-parietal brain regions, the limbic system, and the corpus callosum. Functionally, fMRI analysis discovered significant hypoactivation in several frontal-temporal brain regions, the right postcentral gyrus, the left insula, and the corpus callosum.

**Conclusion:**

This study showed that abnormal alterations in the structure and function of the left superior frontal gyrus and the corpus callosum may be the key brain regions involved in the pathogenesis of ADHD in patients and may be employed as an imaging metric for patients with ADHD pending future research. In addition, this meta-analysis discovered neuroanatomical or functional abnormalities in other brain regions in patients with ADHD as well as findings that can be utilized to guide future research.

## Introduction

According to the Diagnostic and Statistical Manual of Mental Disorders, Fifth Edition (DSM-5) and International Classification of Diseases (ICD-10), attention-deficit/hyperactivity disorder (ADHD) is a widespread neurodevelopmental disorder affected by multiple factors, which are often manifested as inattention and/or impulsiveness hyperactivity (1). The epidemiological investigation showed that over 5% of children and adolescents in the world are impacted by this illness (2), and the morbidity is higher in boys, showing an increasing trend year by year. However, some symptoms of the children could continue into adulthood (3), which would disturb the varied aspects of their learning, family, social lives, and/or physical and psychological health (including a higher proportion of destructive behaviors, anxiety, depressive behaviors, substance use disorders, and so on) (4, 5).

At present, studies in various fields, such as genetics (6, 7), etiology (8), neuropsychology (9), and neurophysiology (10), attempt to explore the pathological mechanisms of ADHD. Similar to other psychiatric and developmental disorders, ADHD has genetic susceptibility (6, 7). Environmental factors (including psychosocial factors, prenatal and perinatal risk factors, nutritional factors, etc.) are also one of the risk factors for ADHD (11, 12). Defects in mood regulation are common in patients with ADHD, which is considered a key factor associated with it (10). Other studies showed that the lack of DA (dopamine) in functional brain areas, such as the cerebral cortex and the striatum, also leads to ADHD (13). In addition, more neuroimaging studies (14–16) focused on patients with ADHD and considered ADHD an important research topic. A large amount of evidence suggests that ADHD has abnormal brain structure and function, although the effect sizes are small.

Voxel-based morphometry is a scientific method used to measure and analyze magnetic resonance imaging (MRI) at the voxel level (17) and reflect anatomical differences *via* quantitatively calculating the differences in density or volume of the gray matter and white matter of each voxel in the MRI. However, most of the current research results are inconsistent. Some studies reported decreased gray matter volume (GMV) in the right and left anterior cingulate cortex (ACC) (18) and the right thalamus (19) in patients with ADHD compared to healthy controls (HCs), while some other studies reported decreased GMV in the frontal lobe, the hippocampus, the temporal cortex, and the occipital cortex using VBM (20). Sutcubasi Kaya et al.'s study (21) reported increased GMV in the precentral gyrus and the supplementary motor area in patients with ADHD. The differences from those of earlier studies are attributed to patients with ADHD having a relatively high average IQ in the current studies. McGrath et al. (22) analyzed the structural neuroimaging data between dyslexia and ADHD, conducted an anatomic likelihood estimate (ALE) meta-analysis on VBM studies of the two diseases, and identified an overlapping region of the right caudate nucleus when using a more relaxed statistical threshold. This suggests that the abnormality of caudate nucleus may be the basis of executive dysfunction.

Functional MRI (fMRI) describes the activation region of neural excitation by monitoring blood oxygen levels and using deoxyhemoglobin as an endogenous contrast agent, characterized by low time resolution and high spatial resolution (23). At present, the fMRI studies of ADHD involve different executive function processes, and different task paradigms can be used to evaluate the same executive function, but there are a lot of heterogeneous research results in these studies (24). Decreased activation in alert task-related brain areas in children with ADHD, including the right frontal-orbital, the superior frontal and bilateral temporoparietal regions, the cerebellum, the hippocampus, the striatum, and the thalamus, was reported in a study by Rubia et al. (25). A meta-analysis of fMRI studies (26) on children with ADHD, with low activation in executive function (frontal-parietal network) and attention (ventral attention network) and high activation in prefrontal and ventral attention networks, identified earlier abnormalities beyond abnormal prefrontal-striatal circuits. The recent meta-analysis (27) of whole-brain fMRI showed reduced activation of the bilateral anterior insula putamen and the globus pallidus in the left inferior frontal gyrus in patients with ADHD during cognitive conversion tasks.

Recently, the results of a combined VBM and fMRI analysis (15) showed that reduced GMV and fMRI underactivation overlapped in the right caudate nucleus during cognitive control in patients with ADHD compared to HCs. Similar studies (28) found that increased GMV and functional activation overlapped in the right fusiform gyrus, while decreased GMV and functional activation overlapped in the right superior temporal gyrus, the left inferior frontal gyrus, and the left postcentral gyrus in patients with ADHD compared to HCs. Samea et al. (29) pooled 96 studies in children and adolescents, and they observed the convergence dysfunction in the left pallidum/putamen for task-fMRI experiments (using neutral stimuli) and decreased activity in the left IFG among different sub-analyses. This study has profoundly elaborated the structural and functional alterations of the brain in patients with ADHD; however, the results remain inconsistent, possibly due to the diversity of sample characteristics, behavioral scales, imaging methods, and statistical analyses, suggesting the need for in-depth investigation to confirm, broaden, and/or amend previous findings.

Based on previous theories and empirical studies in ADHD, we hypothesized that both functional and structural alterations in frontostriatal and fronto-cingulate circuits occur in patients with ADHD relative to HCs. In addition, studies suggest that some symptoms of ADHD in children will gradually get better with age but will not completely disappear. Therefore, we expect that more brain areas in the children group will show changed GMV and abnormal activation compared to the adult group. It may also be associated with delayed maturation of certain brain regions. The objective of our study was to explore the common and specific neural alterations in the structure and function of the brain in ADHD and further explore the differences in brain alterations between adults and children with ADHD. Eventually, the significance of these research results and their prospective development paths are examined and discussed.

## Methods

### Literature search and selection

A comprehensive literature search of fMRI and VBM studies of ADHD from January 2010 to November 2021 was conducted primarily using PubMed, as well as additional searches in the Web of Knowledge and Science Direct databases, with a combination of the following keywords: “voxel-based morphometry” or “VBM” or “morphometry” or “gray matter” or “functional magnetic resonance imaging” or “fMRI” and “ADHD” or “Attention Deficit/Hyperactivity Disorder.” In addition, a manual search was conducted in reference lists of previous meta-analyses. Eligible original studies were identified through reference tracking and consultation to retrieve high-quality meta-analyses and review articles.

Studies were eligible if (1) they involved task-related fMRI or VBM study; (2) they compared patients with ADHD and HCs; (3) the whole-brain outcomes were presented in three-dimensional coordinates (*x, y, z*) for changes in standard stereotactic space (e.g., Talairach space or Montreal Neurological Institute space); (4) the diagnosis of patients with ADHD had to be based on DSM-IV-TR, or DSM-V, or ICD-10; and (5) they were peer-reviewed and published in English as an article. Studies were excluded if (1) the peak coordinates were not reported; (2) VBM was not used and only ROI analyses were used; (3) the patient data were duplicated or no eligible contrasts were found; and (4) neurological or psychiatric comorbidities (such as depression, anxiety, autism, learning disorder, and so on) were found in the patient group. In the case of incomplete or ambiguous information, we will contact the author for clarification.

All the authors evaluated the final inclusion of articles and reached a consensus on all of them before implementing the following steps.

### Statistical analysis

The anisotropic effect size seed-based d mapping (AES-SDM) (version 5.15) meta-analytic software package (https://www.sdmproject.com/) was applied extensively in a recent meta-analysis. We plan to use it to analyze the GMV and functional neural activation changes between patients with ADHD and HCs, following related guidelines (MOOSE) for meta-analyses. In this study, we summarized the AES-SDM data processing procedure. First, the peak coordinates extracted from each dataset were manually imported into the software. Second, the maps of the lower and upper bounds of possible effect sizes were reconstructed for each VBM or fMRI study separately using an anisotropic non-normalized Gaussian kernel. Peak coordinates were converted to Montreal Neurological Institute (MNI) space. After that, this mean analysis was processed, which consists of calculating the mean of the voxel values in the different studies. This mean is weighted by the inverse of the variance and accounts for inter-study heterogeneity. In addition, the results of some studies were *p*-value or *z*-value, which needed to be converted to t-statistics online; full width at half maximum (FWHM) was set to 20 mm because this setting was optimal to balance sensitivity and specificity, and other parameters included voxel *P* = 0.005, peak height *Z* = 1, and cluster extent = 10 voxels (30).

Our research steps are as follows: First, analyses were conducted to examine regional GMV in the patient group relative to HCs using all available data, and fMRI meta-analyses were performed to examine the neural activation abnormalities using the above same method. Second, a conjunction analysis in patient groups relative to HCs was further carried out to examine common areas of structural and functional abnormalities. Then, we divided individuals into two different subtypes based on age group (see **Table 2**) for the next subgroup analysis, namely, adult and pediatric (including adolescents and children) subjects, using a random effects model under the same threshold as before. To examine the effects of age, gender, and IQ, meta-regression analyses were performed. Finally, to assess whether the results were replicable, a jackknife sensitivity analysis was performed, in which the same analysis was repeated in one study, excluding one data at a time. Moreover, Egger's test was used to examine the possible publication bias. A statistical threshold *p*-value of <0.005 was used for all meta-analyses (31, 32), and a decreased threshold *p*-value of <0.0005 and a cluster extent of 20 voxels were applied in the meta-regression to control for false positives (33).

## Results

**Figure 1:**
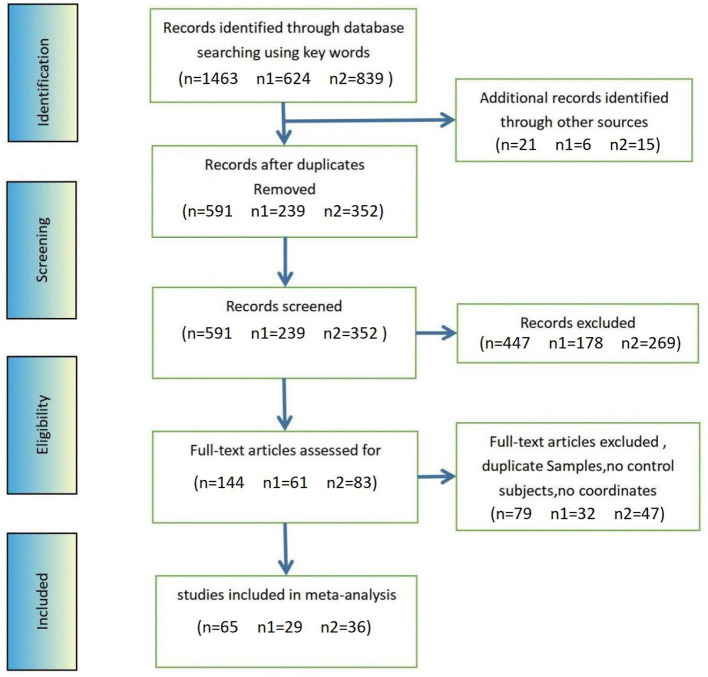
Methods and results of literature search for ADHD fMRI and VBM. *n*, total number of studies; *n*1, number of VBM study; *n*2, number of fMRI study.

A total of 1,463 published papers (624 VBM studies and 839 fMRI studies) were screened, with an increase of 21 documents (six VBM studies and 15 fMRI studies) discovered *via* other sources. After repetitions were eliminated, 591 documents (239 VBM studies and 352 fMRI studies) were reviewed, and 144 full-text publications (61 VBM studies and 83 fMRI studies) were evaluated for eligibility. The final sample included 29 VBM research (1,211 patients with ADHD and 1,032 HCs) and 36 fMRI research (850 patients with ADHD and 813 HCs) studies (see Figure 1; Table 1 for more details).

**Table 1:** Sample characteristics of VBM and fMRI studies.

**References**	**Age group**	**Task**	**Patients**			**Controls**		
			**Number (% male)**	**Mean age (years)**	**IQ**	**Number (% male)**	**Mean age (years)**	**IQ**
**VBM studies in ADHD**								
Ahrendts et al. (34)	Adults	–	31 (65)	31.20	NA	31 (65)	31.50	NA
Montes et al. (35)	Adults	–	20 (50)	28.95	102.9	20 (50)	27.57	100.2
Amico et al. (18)	Adults	–	20 (75)	33.60	NA	20 (75)	34.70	NA
Bonath et al. (36)	Adolescents	–	18 (x)	13.60	106.8	18 (x)	14.10	108.1
Bralten et al. (37)	Adolescents	–	307 (68)	17.06	97.1	196 (49)	16.66	106.6
Tsai et al. (38)	Adolescents	–	118 (83)	11.11	107.4	104 (81)	11.71	108.6
Gehricke et al. (39)	Adults	–	32 (81)	25.31	NA	40 (83)	23.93	NA
He et al. (40)	Children	–	37 (100)	9.90	NA	35 (100)	10.70	NA
Jagger-Rickels et al. (41)	Children	–	41 (x)	9.61	NA	32 (x)	9.66	NA
Kappel et al. (42)	Adults	–	16 (94)	23.50	97.8	20 (100)	23.70	108.4
	Children	–	14 (71)	9.80	104.6	10 (80)	11.00	111.9
Klein et al. (43)	Adults	–	25 (36)	66.90	113.9	34 (18)	68.90	113.2
Kobel et al. (44)	Adolescents	–	14 (x)	10.43	NA	12 (x)	10.92	NA
Kumar and Arya (45)	Children	–	18 (100)	9.60	92.1	18 (100)	9.60	109.7
Li et al. (46)	Adolescents	–	30 (100)	10.30	121.7	30 (100)	10.30	107.1
Lim et al. (47)	Adolescents	–	29 (100)	13.80	92.2	29 (x)	14.40	110.0
Stevens et al. (48)	Adolescents	–	24 (67)	15.70	98.3	24 (70)	16.00	97.4
Moreno-Alcazar et al. (49)	Adults	–	44 (66)	31.61	105.0	44 (66)	32.57	106.0
Ramesh and Rai (50)	Adolescents	–	15 (27)	16.80	>80	15 (27)	16.72	>80
Roman-Urrestarazu et al. (51)	Adults	–	49 (65)	22.23	96.6	34 (57)	22.95	112.2
Sasayama et al. (52)	Adolescents	–	18 (72)	10.60	90.0	17 (71)	10.00	100.0
Seidman et al. (20)	Adults	–	74 (x)	37.30	116.0	54 (x)	34.30	115.8
Sethi et al. (53)	Adults	–	30 (63)	33.70	109.0	30 (63)	32.60	110.1
Shimada et al. (54)	Adolescents	–	17 (88)	10.29	95.3	15 (73)	12.80	104.1
Sutcubasi Kaya et al. (21)	Adolescents	–	19 (74)	10.32	NA	18 (67)	10.17	NA
van Wingen et al. (55)	Adults	–	14 (100)	32.00	104.0	15 (100)	37.00	99.0
Vilgis et al. (56)	Adolescents	–	33 (100)	12.58	92.2	31 (100)	12.75	109.6
Villemonteix et al. (57)	Adolescents	–	38 (58)	10.40	105.7	25 (60)	10.10	109.6
Wang et al. (58)	Adolescents	–	30 (63)	10.60	97.2	25 (48)	10.60	106.6
Zhao et al. (59)	Adolescents	–	36 (x)	12.14	108.8	36 (x)	11.69	121.4
**fMRI studies in ADHD**								
Sáenz et al. (60)	Adolescents	Stop	18 (67)	10.30	102.6	14 (64)	11.12	121.5
Bédard et al. (61)	Adolescents	*N*-back	24 (87)	13.07	110.0	21 (76)	12.44	111.1
Chantiluke et al. (62)	Adolescents	Stop	18 (100)	14.30	95.0	25 (100)	13.40	109.0
Chen et al. (63)	Adults	Go-NoGo	29 (100)	24.90	NA	25 (100)	25.64	NA
Christakou et al. (64)	Adolescents	SAT	20 (100)	14.00	108.2	20 (100)	14.70	114.0
Cubillo et al. (65)	Adults	Simon	11 (100)	29.00	92.0	15 (100)	28.00	112.0
Cubillo et al. (66)	Adolescents	Stop	19 (100)	13.00	92.0	29 (100)	13.00	110.0
Cubillo et al. (67)	Adults	Stop/switch	11 (100)	29.00	92.0	14 (100)	28.00	106.0
Dibbets et al. (68)	Adults	Switch	15 (100)	28.90	NA	14 (100)	28.80	NA
Fan et al. (69)	Adolescents	Stroop	27 (89)	12.10	105.2	27 (78)	11.80	110.4
Fan et al. (70)	Adolescents	Stroop	25 (92)	10.90	107.2	23 (91)	11.20	109.1
Janssen et al. (71)	Adolescents	Stop	21 (90)	10.60	98.6	17 (76)	10.28	108.7
Kooistra et al. (72)	Adults	Go-NoGo	11 (100)	21.50	110.0	11 (100)	22.30	125.0
Ma et al. (73)	Children	Go-NoGo	15 (53)	9.82	100.2	15 (53)	9.91	102.6
Ma et al. (74)	Adolescents	Stroop	25 (76)	15.40	98.3	33 (67)	15.30	108.9
Materna et al. (75)	Adults	Stimuli	30 (63)	31.40	NA	35 (54)	28.89	NA
Mehren et al. (76)	Adults	Go-NoGo	20 (100)	31.40	NA	20 (100)	29.50	NA
Passarotti et al. (77)	Adolescents	Stop	11 (55)	13.09	101.2	15 (47)	14.13	107.6
Dibbets et al. (78)	Adults	Go-NoGo	15 (100)	28.90	NA	13 (100)	28.10	NA
Pretus et al. (79)	Adults	ITIs	21 (52)	36.50	NA	24 (50)	34.33	NA
Rasmussen et al. (80)	Adults	Go/No-Go	25 (68)	25.00	106.3	12 (50)	24.10	107.1
Rubia et al. (81)	Adolescents	Simon	12 (100)	13.00	90.0	13 (100)	13.00	102.0
Rubia et al. (82)	Adolescents	Stop	12 (100)	13.00	91.0	13 (100)	13.00	100.0
Schulz et al. (83)	Adults	Go-NoGo	14 (100)	23.30	NA	14 (100)	22.80	NA
Sebastian et al. (84)	Adults	Go-NoGo/Stroop	20 (55)	33.30	115.3	24 (46)	30.30	115.7
Shang et al. (85)	Adults	Stroop	25 (56)	28.50	113.1	30 (50)	28.17	115.4
Siniatchkin et al. (86)	Children	Go-NoGo	17 (82)	9.30	NA	14 (71)	9.10	NA
Spinelli et al. (87)	Adolescents	Go-NoGo	13 (69)	10.60	109.2	17 (47)	10.50	108.8
Tamm and Juranek (88)	Children	FRT	12 (54)	9.00	>84	10 (80)	10.00	>84
Tegelbeckers et al. (89)	Adolescents	MVOT	19 (100)	13.32	104.8	19 (100)	13.58	108.63
Thornton et al. (90)	Adolescents	Go-NoGo	20 (90)	12.40	109.7	20 (40)	10.55	112.6
van Rooij et al. (91)	Adolescents	Stop	185 (70)	17.30	95.3	124 (44)	16.50	107.1
Vetter er al. (92)	Adolescents	PDT	25 (100)	14.26	106.0	25 (100)	14.02	110.0
Wang et al. (93)	Children	Go-NoGo/Lure	28 (89)	9.60	105.4	31 (52)	10.23	108.2
Yang et al. (94)	Adults	IGT	20 (45)	26.90	NA	20 (40)	27.70	NA
Zamorano et al. (95)	Adolescents	Stroop	17 (100)	11.60	104.2	17 (100)	11.70	109.8

NA: Not applicable. It indicates that the data is not applicable.

The Wilcoxon *W*-tests in the whole-group VBM analysis indicated that patient groups did not differ in age (*z* = −0.325; *P* = 0.745), and the patient groups have a slightly lower IQ than the control groups (*z* = −2.653; *P* = 0.008). The chi-squared test revealed that both groups had a significantly higher proportion of boys/men (χ^2^ = 8.450; *P* = 0.004). In the whole-group fMRI meta-analysis, patient groups did not differ in age (*z* = −1.077; *P* = 0.282), and the patient groups have a slightly lower IQ than the control groups (*z* = −3.616; *P* < 0.001), but a higher percentage of patients with ADHD were boys/men (χ^2^ = 50.655; *P* < 0.001). Details of the regression analysis results of the subgroups are shown in Table 2. As a result, age, IQ, and sex were included as covariates in all between-group meta-analyses performed including only these studies which were age-, IQ-, and sex-matched (see Table 2 for more details).

**Table 2:** Demographic information for studies included in the meta-analysis.

**Characteristic**	**The whole group**				**Adult group**				**Pediatric group**			
	**ADHD**	**Hc**	***z*****/**χ^2^	***P***	**ADHD**	**Hc**	***z*****/**χ^2^	***P***	**ADHD**	**Hc**	***z*****/**χ^2^	***P***
**VBM**												
Patients, *n*.	1,211	1,032	–	–	355	342	–	–	856	690	–	–
Male sex, *n*. (%)	954 (79)	759 (74)	8.45	0.004	263 (74)	240 (70)	1.325	0.25	691 (81)	519 (75)	6.811	0.009
Mean age, y	19.70 ± 12.83	19.99 ± 13.04	−0.325	0.745	33.30 ± 12.06	33.61 ± 12.68	−0.295	0.797	11.82 ± 2.44	12.09 ± 2.38	−0.511	0.609
Mean FSIQ (S.D)	102.48 ± 8.42	107.98 ± 5.60	−2.653	0.008	105.65 ± 6.97	108.11 ± 6.04	−0.735	0.505	100.67 ± 8.87	107.91 ± 5.57	−2.666	0.008
Max/min age, y	66.9/9.6	68.9/9.6	–	–								
Max/min FSIQ (S.D)	121.7/90.0	121.4/100.2	–	–								
**fMRI**												
Patients, *n*.	850	813	–	–	267	271	–	–	583	542	–	–
Male sex, *n*. (%)	728 (86)	580 (71)	50.665	<0.001	207 (77)	197 (73)	1.681	0.195	521 (89)	383 (71)	62.229	<0.001
Mean age, y	18.57 ± 8.53	18.22 ± 7.98	−1.077	0.282	28.46 ± 4.00	27.62 ± 3.13	−0.919	0.376	12.27 ± 2.12	12.25 ± 1.98	−0.035	0.972
Mean FSIQ (S.D)	102.42 ± 7.37	110.05 ± 5.35	−3.616	<0.001	104.78 ± 10.35	113.53 ± 6.93	−1.444	0.18	101.71 ± 6.40	109.04 ± 4.59	−3.555	<0.001
Max/min age, y	36.5/9.0	34.33/9.1	–	–								
Max/min FSIQ (S.D)	115.3/90.0	125.0/100.0	–	–								

### Regional differences in GMV and fMRI by meta-analysis

#### ADHD VBM

**Figure 2:**
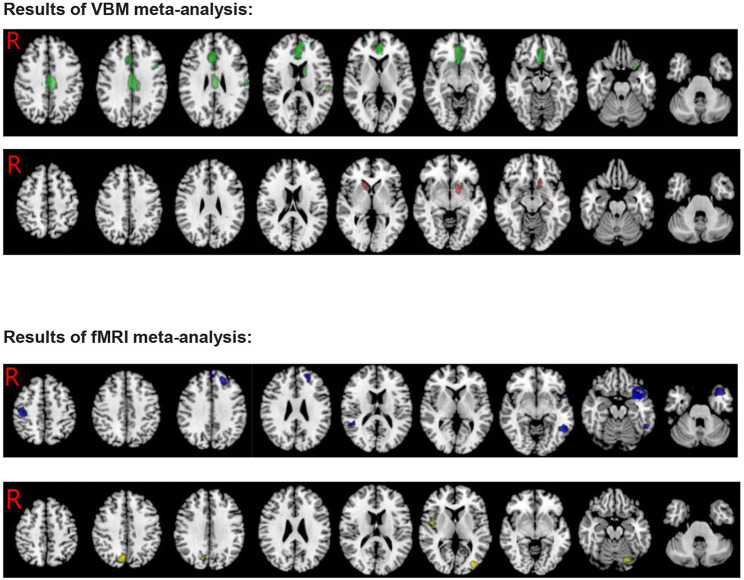
Results of VBM and fMRI meta-analysis for ADHD (Green, GMV decreased in ADHD; Red, GMV increased in ADHD; Blue, hypoactivation in ADHD; Yellow, overactivation in ADHD).

Patients with ADHD showed significantly lower GMV in the bilateral anterior cingulate cortex/median cingulate/superior frontal gyrus/olfactory cortex, the left precentral/postcentral gyrus, the left inferior frontal gyrus opercular/orbital part, the left supramarginal gyrus, the left caudate nucleus, the left anterior thalamic projections, the right gyrus rectus, and the corpus callosum compared to HCs. In contrast, the results also showed an increase in the left striatum/lenticular nucleus, the right caudate nucleus, and the right anterior thalamic projections (see Table 3; Figure 2).

**Table 3:** Meta-analysis results for voxel-based morphometry and fMRI studies in ADHD.

**Contrast**	**Anatomical region**	**MNI coordinates**			**SDM *Z*-score**	***P*-value**	**Number of voxels**	**Clusters'breakdown (number of voxels)**	**Jack-knife sensitivity**
		***X***	***Y***	***Z***					
**VBM RESULTS**									
**ADHD**<**control**									
	R anterior cingulate	2	30	−6	−2.449	0.000221908	2,557	L anterior cingulate (BA 32, 24, 10, 11, 25) (682)	22 out of 30
								L median cingulate (BA 23, 24) (354)	
								R superior frontal gyrus (BA 11, 10) (169)	
								L median network (121)	
								R median cingulate (BA 23, 24, 32) (276)	
								callosum (117)	
								R anterior cingulate (BA 32, 24, 11, 10, 25) (311)	
								L superior frontal gyrus (BA 32, 10, 24, 11, 10) (212)	
								R median network (54)	
								R gyrus rectus (BA 11) (43)	
								(74)	
	L postcentral gyrus	−58	−18	18	−1.883	0.002394617	135	L postcentral gyrus (BA 48, 43) (100)	27 out of 30
								L supramarginal gyrus (BA 48, 2) (25)	
	L caudate nucleus	−10	4	16	−1.965	0.001656592	97	L anterior thalamic projections (49)	25 out of 30
								L caudate nucleus (46)	
	L inferior frontal gyrus, opercular part	−46	14	32	−1.934	0.001904368	52	L precentral gyrus (BA 44) (21)	26 out of 30
								L inferior frontal gyrus (BA 48, 44) (31)	
	L inferior frontal gyrus, orbital part	−26	16	−24	−2.083	0.000985742	39	L inferior frontal gyrus (BA 38) (35)	29 out of 30
	R superior frontal gyrus, dorsolateral	28	66	2	−1.994	0.001486301	34	R superior frontal gyrus (BA 11, 10) (34)	27 out of 30
**ADHD** > **control**									
	L striatum	−16	16	−6	1.334	0.000129044	188	L striatum (121)	28 out of 30
								L lenticular nucleus (BA 25, 48) (25)	
	R caudate nucleus	12	22	2	1.062	0.000830889	103	R anterior thalamic projections (33)	27 out of 30
								R caudate nucleus (BA 25) (46)	
**fMRI RESULTS**									
**ADHD**<**control**									
	L superior temporal gyrus	−34	16	−24	−3.177	0.000005186	752	L superior temporal gyrus (BA 38, 48, 20, 21, 34) (430)	35 out of 36
								L inferior frontal gyrus (BA 38, 47) (112)	
								L insula (BA 48, 38, 47) (106)	
								L middle temporal gyrus (BA 38) (23)	
								(46)	
	R postcentral gyrus	40	−22	52	−2.156	0.002084970	259	R precentral gyrus (BA 4, 6, 3) (174)	33 out of 36
								R postcentral gyrus (BA 3, 4, 6) (82)	
	callosum	−50	−52	−8	−2.784	0.000170290	229	L inferior temporal gyrus (BA 37, 20, 21) (149)	36 out of 36
								L middle temporal gyrus (BA 20, 37, 21) (31)	
	L middle frontal gyrus	−22	42	34	−2.981	0.000020623	211	L superior frontal gyrus, dorsolateral (BA 9) (72)	35 out of 36
								L middle frontal gyrus (BA9, 46) (110)	
								callosum (28)	
	R arcuate network, posterior segment	44	−44	14	−2.624	0.000376761	75	R arcuate network, posterior segment (19)	33 out of 36
								R superior temporal gyrus (BA 42, 41, 22) (41)	
	L superior frontal gyrus, medial	−6	54	36	−2.411	0.000939250	71	L superior frontal gyrus, medial (BA 9, 10) (69)	35 out of 36
**ADHD** > **control**									
	L middle occipital gyrus	−36	−84	10	1.410	0.000588357	347	L middle occipital gyrus (BA 19, 18, 39) (309)	34 out of 36
	R insula	50	10	−8	1.305	0.001083791	334	R insula (BA 48, 38) (166)	31 out of 36
								(40)	
	R precuneus	8	−72	40	1.263	0.001414061	199	R precuneus (BA 7, 19) (126)	32 out of 36
								R cuneus cortex (BA 7, 19) (46)	
	L cerebellum, hemispheric lobule VI	−20	−76	−16	1.211	0.001909494	82	L cerebellum (BA 18) (34)	32 out of 36
								L fusiform gyrus (BA 18) (23)	

#### ADHD fMRI

Patients with ADHD showed overactivation in the middle occipital gyrus, the right insula, the right precuneus cortex, the left cerebellum hemispheric lobule, and the left fusiform gyrus compared to HCs. In contrast, the results also showed hypoactivation in the bilateral superior temporal gyrus, the left middle/inferior temporal gyrus, the left superior/middle/inferior frontal gyrus, the right precentral /postcentral gyrus, the left insula, and the corpus callosum (see Table 3; Figure 2).

### Multimodal VBM and fMRI analyses

**Figure 3:**
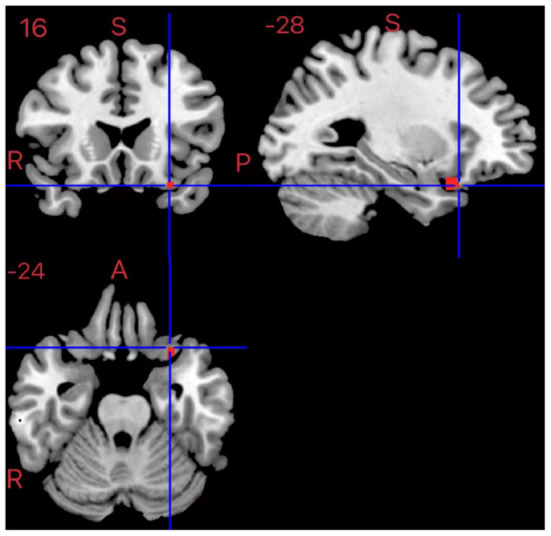
Results of multimodal VBM and fMRI for ADHD.

In patients with ADHD, there was an overlap between decreased GMV in the left inferior frontal gyrus and hypoactivation compared to HCs (MNI coordinates, −28, 16, −24; 17 voxels) (see Figure 3).

### Subgroup analysis

#### ADHD VBM

**Figure 4:**
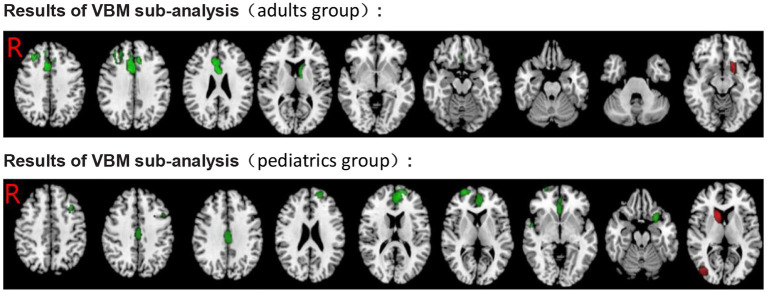
Results of subgroup analysis of VBM for ADHD. Green, GMV decreased in ADHD; Red, GMV increased in ADHD.

Individuals were divided into two independent subgroups, namely, adult and pediatric groups (including adolescents and children). The findings of the adult group's (11 datasets) analysis were substantially in line with the pooled analysis, except for the additional significant GMV decrease in the right middle frontal gyrus. In addition, relative to the pooled analysis, no significant GMV decrease regions were found in the left supramarginal gyrus and the left inferior frontal gyrus. Similarly, the results also indicated increased GMV in the left striatum and the left lenticular nucleus. The results of the pediatric group's (19 datasets) analysis showed GMV decrease in additional brain regions—the bilateral middle frontal gyrus, the bilateral superior temporal gyrus, and the left insula. Similarly, relative to the pooled analysis, no significant GMV decrease regions were found in the left supramarginal gyrus, the left caudate nucleus, and the left anterior thalamic projections, the left precentral /postcentral gyrus, and the right gyrus rectus. In addition, the results showed increased GMV in the left striatum, the left middle occipital gyrus, the right caudate nucleus, and the right anterior thalamic projections (see Tables 4, 5; Figure 4).

**Table 4:** Subgroup analysis results for voxel-based morphometry and fMRI studies in ADHD (adults group).

**Contrast**	**Anatomical region**	**MNI coordinates**			**SDM *Z*-score**	***P*-value**	**Number of voxels**	**Clusters'breakdown (number of voxels)**
		***X***	***Y***	***Z***				
**VBM RESULTS**								
**ADHD**<**control**								
	R median cingulate, paracingulate gyri	12	28	30	−2.216	0.000252903	793	R median cingulate (BA 32, 24) (303)
								R anterior cingulate (BA 24, 32) (167)
								L anterior cingulate (BA 24) (111)
								L median cingulate (BA 24) (79)
								R median network, cingulum (60)
								Corpus callosum (36)
	L anterior thalamic projections	−12	−2	10	−2.259	0.000196099	187	L anterior thalamic projections (87)
								L caudate nucleus (91)
	R superior frontal gyrus, dorsolateral	22	38	36	−1.955	0.001073420	153	R middle frontal gyrus (BA 9, 46) (91)
								Corpus callosum (32)
								R superior frontal gyrus, dorsolateral (BA 9) (30)
	L median network, cingulum	−10	26	34	−2.092	0.000454128	68	L median network, cingulum (21)
								L superior frontal gyrus, medial (BA 32) (20)
	R gyrus rectus	4	30	−18	−1.825	0.002740383	53	R gyrus rectus (BA 11) (14)
**ADHD** > **control**								
	L inferior network, uncinate fasciculus	−18	18	−12	1.167	0.002544284	65	L striatum (19)
								L lenticular nucleus (BA 48) (17)
**fMRI RESULTS**								
**ADHD**<**control**								
	L lenticular nucleus, putamen	−24	4	−6	−1.757	0.000180602	717	L lenticular nucleus (BA 48, 11, 34) (283)
								L striatum (156)
								L insula (BA 48) (28)
								Undefined (BA 48, 34) (183)
	R insula	34	22	2	−1.783	0.000165164	280	R insula (BA 47, 48) (271)
	R arcuate network	56	−38	16	−1.428	0.001274705	269	R superior temporal gyrus (BA 42, 48) (167)
								Corpus callosum (25)
	R caudate nucleus	14	24	0	−1.524	0.000717342	94	R anterior thalamic projections (35)
								R caudate nucleus (BA 25) (35)
	Corpus callosum	−12	−28	58	−1.424	0.001326323	60	Corpus callosum (50)
	R postcentral gyrus	32	−28	58	−1.328	0.00248754	44	R precentral gyrus (BA 4, 3) (17)
								R postcentral gyrus (BA 6, 4, 3) (19)
**ADHD** > **control**								
	R middle temporal gyrus	44	−72	20	1.039	0.002172709	186	L middle occipital gyrus (BA 39, 19) (122)
								R middle temporal gyrus (BA 39) (40)

**Table 5:** Subgroup analysis results for voxel-based morphometry and fMRI studies in ADHD (pediatrics group).

**Contrast**	**Anatomical region**	**MNI coordinates**			**SDM *Z*-score**	***P*-value**	**Number of voxels**	**Clusters'breakdown (number of voxels)**
		***X***	***Y***	***Z***				
**VBM RESULTS**								
**ADHD**<**control**								
	L superior frontal gyrus, medial	−10	50	14	−2.779	0.000056744	1,119	L anterior cingulate, paracingulate gyri (BA 32, 10, 11) (362)
								L superior frontal gyrus, medial (BA 10, 32) (284)
								L superior frontal gyrus, dorsolateral (BA 10, 46) (123)
								R anterior cingulate, paracingulate gyri (BA 11, 32, 10) (114)
								R superior frontal gyrus, medial (BA 10, 11) (46)
								Corpus callosum (113)
	L median cingulate/paracingulate gyri	0	−20	36	−1.824	0.001692772	370	L median cingulate/paracingulate gyri (BA 23) (170)
								R median cingulate/paracingulate gyri (BA 23) (100)
								L median network, cingulum (49)
	R superior frontal gyrus, dorsolateral	28	66	2	−2.411	0.000175476	260	R superior frontal gyrus, dorsolateral (BA 11, 10) (192)
								R superior frontal gyrus, orbital part (BA 11) (27)
								R middle frontal gyrus (BA 10) (24)
	L inferior frontal gyrus, orbital part	−26	16	−24	−2.518	0.000149667	234	L inferior frontal gyrus, orbital part (BA 38, 11) (110)
								L superior temporal gyrus (BA 38) (69)
								L insula (BA 48, 47) (38)
	R superior temporal gyrus	52	4	−8	−1.572	0.003648698	61	R superior temporal gyrus (BA 38, 21) (57)
	L middle frontal gyrus	−36	16	48	−1.777	0.002043664	31	L middle frontal gyrus (BA 9) (24)
	L middle frontal gyrus	−46	12	38	−1.693	0.002564907	25	L middle frontal gyrus (BA 44) (20)
**ADHD** > **control**								
	R caudate nucleus	12	20	2	1.622	0.000000000	468	R anterior thalamic projections (168)
								R caudate nucleus (BA 25) (203)
								L striatum (32)
	L middle occipital gyrus	38	−78	2	1.182	0.000082552	351	L middle occipital gyrus (BA 19, 18) (246)
**fMRI RESULTS**								
**ADHD**<**control**								
	L superior temporal gyrus	−34	14	−26	−3.087	0.000005186	503	L superior temporal gyrus (BA 38, 20) (307)
								L inferior frontal gyrus, orbital part (BA 38, 47) (67)
								L insula (BA 48, 38) (45)
								L middle temporal gyrus (BA 38) (25)
	L inferior temporal gyrus	−48	−54	−8	−2.839	0.000072241	258	L inferior temporal gyrus (BA 37, 20) (158)
								L middle temporal gyrus (BA 37, 20) (53)
								L inferior network, inferior longitudinal fasciculus (24)
	L middle frontal gyrus	−24	40	34	−2.902	0.000030994	223	L superior frontal gyrus, dorsolateral (BA 9) (80)
								L middle frontal gyrus (BA 46, 9) (119)
								Corpus callosum (24)
	L superior frontal gyrus, medial	−8	54	38	−2.308	0.001346946	61	L superior frontal gyrus, medial (BA 9, 10) (52)
	R precentral gyrus	40	−20	54	−1.933	0.003581583	62	R precentral gyrus (BA 4, 6) (52)
	R arcuate network, posterior segment	44	−44	16	−2.492	0.000645101	40	R arcuate network, posterior segment (17)
	Corpus callosum	−12	38	30	−2.569	0.000433505	22	Corpus callosum (18)
**ADHD** > **control**								
	R insula	48	12	−6	1.751	0.000211596	914	R insula (BA 48, 47, 38) (493)
	L middle occipital gyrus	−42	−80	−8	1.352	0.002601027	136	L middle occipital gyrus (BA 19) (46)
								L cerebellum, hemispheric lobule VI (BA 18) (32)
								L fusiform gyrus (BA 18, 19) (49)
	L precuneus	−6	−64	52	1.314	0.003158391	33	L precuneus (BA 7) (33)
	R fusiform gyrus	24	−76	−14	1.319	0.003096461	21	R fusiform gyrus (BA 18) (19)

#### ADHD fMRI

**Figure 5:**
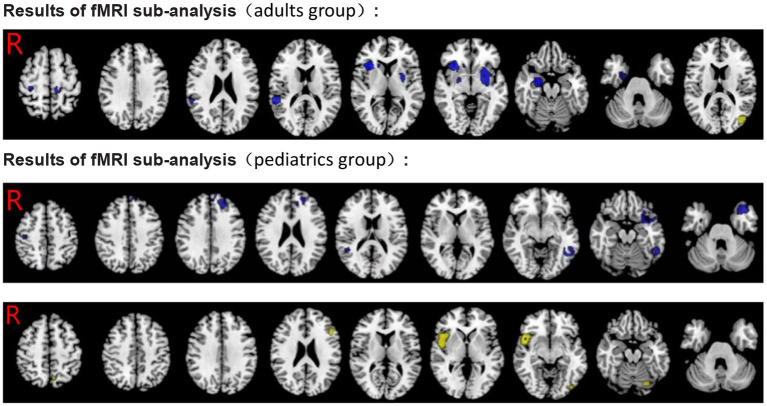
Results of subgroup analysis of fMRI for ADHD. Blue, hypoactivation in ADHD; Yellow, overactivation in ADHD.

Similarl to the previous analysis, we divided individuals into adults and pediatrics (including adolescents and children), two independent subgroups. The findings of the adult group (11 datasets) were partly consistent with the pooled analysis, and significant hypoactivation in the bilateral insula, the left striatum, the left lenticular nucleus, the right superior temporal gyrus, the right precentral/postcentral gyrus, the right caudate nucleus, the right anterior thalamic projections, and the corpus callosum was found. In addition, significant overactivation regions were found in the left middle occipital gyrus and the right middle temporal gyrus. Relative to the pooled analysis, in the pediatric group (22 datasets), no significant hypoactivation was found in the right superior temporal gyrus and the right postcentral gyrus. On the contrary, overactivation of the right fusiform gyrus was found. The rest was largely consistent with the pooled analysis (see Tables 4, 5; Figure 5).

### Publication bias

Egger's tests were conducted to investigate the potential publication bias. The results of the Egger's tests were non-significant (*P* > 0.05 for all comparisons, Bonferroni corrected), indicating that there was no publication bias. Jackknife reliability analyses revealed that the findings were robust in terms of disorder differentiation.

## Discussion

The purpose of our meta-analysis was to investigate the altered GMVs and functional happenings in patients with ADHD and further explore the differences in brain alterations between adults and children with ADHD. Our meta-analysis of structural studies showed that the largest GMV reductions in patients with ADHD relative to HCs were in several frontal parietal brain regions, the limbic system, and the corpus callosum. In addition, we also found that the GMV increased in some brain areas. In fMRI, our study discovered that patients with ADHD relative to HCs showed significant hypoactivation in several frontal-temporal brain regions, the right postcentral gyrus, the left insula, and the corpus callosum. Conversely, we observed overactivation in the middle occipital gyrus, the right insula, the right precuneus cortex, and the left cerebellum hemispheric lobule. Most of the brain region alterations we found were consistent with previous literature, suggesting that the results of the meta-analysis using the seed-based d mapping software package were credible. The results of the subgroup analysis further explored and supported the above results. Moreover, subgroup analysis found relatively more brain areas with reduced GMV and abnormal activation in children and adolescents relative to adults. This makes it clear that patients with ADHD may be associated with delayed maturation of brain development.

In recent years, ADHD has often been considered as related to brain changes in the frontal region. Earlier studies discovered reduced volume in the right superior frontal gyrus (96) and the bilateral dorsolateral prefrontal (97). Bralten et al.'s (37) VBM analysis mentioned that ADHD has a link with reduced GMV in the frontal and precentral regions (including the left precentral gyrus, the medial and left orbitofrontal cortices, the frontal pole, the paracingulate and cingulate cortices), concerning decision-making, executive function, and motor function areas. This is largely consistent with the results of our meta-analysis. In fMRI, we found hypoactivation in the left superior middle and the inferior frontal gyrus and the right anterior central gyrus. Task-based fMRI studies on patients with ADHD revealed inadequate activity in the frontal lobe and striatum (64, 98). Regarding previous fMRI studies, patients with ADHD had lower activation for inhibition in the supplementary motor area, the anterior cingulate cortex, and the dorsolateral prefrontal cortex compared to HCs (99, 100). It is of great importance for the orbitofrontal cortex to regulate emotion and impulsive behavior, and impairment in this area may be the cause of impulsivity in ADHD (101). Moreover, previous findings suggested that the prefrontal cortex and its connections may be closely related to ADHD symptoms including inattention, obliviousness, impulsivity, poor planning, and hyperaction in patients with ADHD (102). In addition, our multimodal results show a decrease in the volume of the left subfrontal gyrus gray matter and a decrease in activity. Thus, our findings support the frontal-striatal circuit theory of ADHD and confirm prior VBM and fMRI studies on ADHD. Future longitudinal studies should strive to evaluate the long-term consequences of ADHD treatments on prefrontal volume and function to confirm the existing results.

The present findings also involve the limbic system-associated brain area. Because of its importance in symptom severity and emotional regulation, the anterior cingulate cortex has long been a focus of ADHD study (103). Similarly, we also found that volume reductions in the bilateral anterior cingulate/paracingulate gyrus, the bilateral median cingulate, the bilateral olfactory cortex, and the left caudate nucleus. However, in the present pediatric group of subgroup analysis, we found no significant GMV decrease in the left caudate nucleus. In a study of 24 adolescent subjects with ADHD vs. HCs, the ADHD group had a lower mean (left and right) caudate and putamen volumes (104). The article of Montes et al. (35) suggested that patients with ADHD had a smaller GMV and a lower concentration of gray matter in the right caudate compared to HCs, both in the overall sample and within each sex. Moreover, a previous study (40) found reduced GMVs in the right orbitofrontal cortex, the left anterior cingulate cortex, and the left posterior midcingulate cortex in patients with ADHD. Amico et al. (18) found significantly smaller GMV in the right and left anterior cingulate cortices but no GMV difference in the PFC, the hippocampus, and the amygdala. Bonath et al. (36) discovered substantially lower GMV in participants with ADHD relative to their matched controls within the anterior cingulate cortex, within the bilateral hippocampus/amygdala, and in broad cerebellar areas. Further, reduced anterior cingulate cortex GMV was also observed to connect with grades of selective inattention. However, these alterations in the hippocampus, the amygdala, and the cerebellum did not appear in our study-based VBM, indicating that they are less consistent across samples compared to the changes in the limbic system. Conversely, we also found that the GMV increased in the left striatum/lenticular nucleus and the right caudate nucleus. Collectively, the limbic system should receive more attention in future research to explore the possible pathogenesis of ADHD.

In patients with ADHD, inattention was linked to reduced GMV not only in the frontal and anterior cingulate but also in the parietal regions. Although only a few ADHD functional MRI investigations have focused on the parietal cortex, it has been recognized as exhibiting changed function in ADHD. This can also be observed in our study, whose results showed that theparietal lobes (including the left postcentral gyrus and left supramarginal gyrus) are smaller in size in patients with ADHD. Simultaneously, we also found significant hypoactivation in the right postcentral gyrus. Previous research (105) found that patients with ADHD doing a visual oddball task showed considerably reduced parietal cortex activation, including the superior parietal gyrus and numerous regions of the inferior parietal lobe. Another study (106) indicated that, in participants with ADHD conducting a spatial working memory mental rotation assignment, the inferior parietal lobe activation was significantly lower, as well as parieto-occipital and caudate activation. Prior research (64) found that successful inhibition activated a cluster of the right angular gyrus, the intraparietal sulcus, and the inferior parietal gyrus in the ADHD group. These regions have been associated with action cancellation and action withholding (107). A previous meta-analysis (41) revealed that smaller GMV was found in the bilateral inferior parietal lobule in subjects with ADHD. However, Seidman et al. (20) found that adults with ADHD had significantly smaller GMVs in the inferior parietal cortex, relative to HCs. We speculated that this difference may be related to the maturational delay or sample diversity. The structural and functional abnormalities in the parietal lobes of those with ADHD further support the conclusion that the parietal cortex plays a role in the pathogenesis of ADHD in patients.

In addition, our analysis results suggest that the temporal lobe may play a crucial role in ADHD. The results of the meta-analysis of fMRI indicate significant hypoactivation in the bilateral superior temporal gyrus and left middle/inferior temporal gyrus. However, decreased GMV was found in the bilateral superior temporal gyrus of the pediatric group alone. Sowell et al. (97) found reduced GMV bilaterally in the lateral aspects of anterior and midtemporal cortices and increased GMV in the more posterior aspects of the temporal lobes bilaterally. The temporal lobe includes auditory language-related centers, while some children with ADHD have abnormal auditory language functions. Using VBM, the studies of Kobel et al. (44) detected isolated areas in right temporal regions where boys with ADHD displayed decreased GMV, demonstrating some type of impairment to temporal brain matter due to ADHD. This result was further supported by a prior investigation (108) where the authors discovered a link between damaged inhibitory control and decreases in medial temporal gray matter. Some studies (91, 109) found insufficient activation in the left superior temporal gyrus. Although these temporal lobe abnormalities in the brain are not completely consistent, we reasoned that the temporal lobe plays a key role in the development of ADHD. As a result, we came to an agreement that research on ADHD ought not to be limited to the frontostriatal deficit hypothesis but also expanded the range to less studied brain areas like the temporal lobe.

It is worth mentioning that we simultaneously found reduced corpus callosum GMV in patients with ADHD relative to HCs and showed hypoactivation in the corpus callosum in fMRI. Considering the anterior or genu region of the corpus callosum connects homologous areas of the frontal cortex (110), theories concerning ADHD being a frontal-striatal illness (111) have motivated some scholars to anticipate that the anterior or genu region of the corpus callosum would be influenced. Significantly smaller areas have been reported in the total corpus callosum area and splenium (112). The meta-analysis of Valera et al. (113) also showed that the splenium located in the posterior region of the corpus callosum is smaller in individuals with ADHD. In patients with ADHD, there is evidence that there are sex disparities in the morphology and white matter integrity of the corpus callosum. For example, female patients with ADHD distinctly had a smaller splenium than male patients with ADHD, according to a meta-analysis (114), while male children and adolescents with ADHD have a smaller anterior corpus callosum. Dramsdahl et al. (115) discovered lower fractional anisotropy values in the isthmus/splenium of the corpus callosum in the ADHD group, and subsequent investigation revealed that FA (fractional anisotropy) values in the posterior part of the corpus callosum were considerably lower in women than in men. These research results show that the role of the corpus callosum in ADHD should be further explored.

The meta-analysis of Jagger-Rickels et al. (41) found reduced left and right insula volume in the ADHD group. Furthermore, in a previous large meta-analysis (16) of sMRI and fMRI studies in children and adults with ADHD, the anterior insula was the only region with both structural and functional abnormalities. However, we found no statistically significant GMV change in the insula. However, overactivation was found in the right insula among patients with ADHD in our study, and this finding is consistent with the results of a previous study (80). In contrast, we also found hypoactivation in the left insula. The insula is also a critical neural structure in the emotional response during decision-making (116). Vetter et al. (92) demonstrated that male patients with ADHD who did not have oppositional defiant disorder/conduct disorder had hyperactivation of the anterior insula for negative stimuli, and hyperactivation of the anterior insula is the basis of this emotional lability.

In our study, increased activation was also found in the left middle occipital gyrus, right precuneus, and left cerebellar hemisphere among patients with ADHD compared to HCs. Occipital cortex, as the center of visual cortex, is responsible for the processing of visual information. To maintain attention, occipital cortex interacts with the dorsal attention network (117), inhibiting the attention caused by irrelevant stimuli (118), whereas the attention caused by hardly ignoring irrelevant stimuli is one of the main symptoms of ADHD. The precuneus is related to visual imagery, attention, and memory retrieval by engaging in the visual process and integrating relevant memory (119). A possible explanation is that high activation in the left middle occipital gyrus and the right precuneus is associated with the severity of symptoms of impulsive craving in patients with ADHD. Although many studies reported abnormalities of structure and function in the cerebellar in patients with ADHD, our study did not reveal its structural differences but found that the left cerebellar hemisphere showed high activation on the task-state fMRI. A recent study (120) suggests that inadequate neuromodulation of prefrontal-cerebellar circuits may underlie cognitive deficits in psychiatric disorders (including schizophrenia, bipolar disorder, and ADHD). In addition, studies (121) showed that both MPH and ATX can restore abnormal brain function in ADHD, mainly involving fronto-cingulo-parieto-cerebellum circuit. In conclusion, the brain changes of patients with ADHD may not be influenced by a single brain region but by multi-channel connections or there may be a certain brain region that is the most critical, which needs to be further studied by other methods such as brain structural network covariation and functional connectivity in the future.

The main results of the whole-group analysis were also found in the adult group and the pediatric group, which further supported the whole-group results. Alterations in the left superior frontal gyrus and the corpus callosum persisted in the pediatrics group and adult group, which we believe further illustrates the crux of this brain region. Subgroup analysis found relatively more brain areas with reduced GMV and abnormal activation in children and adolescents compared to adults. Longitudinal MRI studies (122, 123) have already shown that structural differences in the frontal, striatal, parietal, and cerebellar regions of children with ADHD could be caused by a delay in structural maturation. The research of Hoogman et al. (124) proposed a related model of ADHD brain maturation delay disorder and found a peak delay in subcortical volume maturation. The pediatric group result in our study found that both the left insula and the left superior temporal gyrus reduced GMV in the VBM meta-analysis and hypoactivation in the fMRI meta-analysis, and no such finding was observed in the adult group. The left insula and the left superior temporal gyrus brain area were changed in the pediatric group, but they did not change in the adult group, and we could consider that the alterations in these brain areas may result from delayed maturation. One study showed that many adult patients with ADHD have improved symptoms compared to childhood (125). In addition, evidence reveals that the symptoms of ADHD, as well as ADHD diagnosis, are linked to substantial alterations in the macroscopic and microscopic structures of the brain. Therefore, we speculate that abnormal structure and function of some brain regions in patients with ADHD may also be gradually normal with increasing age, and these brain regions are caused by delayed maturation disorder, which still needs further research to prove.

## Limitation

Our study has several limitations that are inherent in all meta-analyses. In the first place, since it was based predominantly on the peak coordinates of published studies, instead of raw statistical brain maps, it may be underpowered to identify some results with small or moderate effect sizes. Second, for the many papers included in this meta-analysis, different statistical thresholds may have been applied. Additionally, the heterogeneity of the methodologies among VBM and fMRI studies could not be prevented, such as the differences in MRI machines, smoothing kernel size, statistical thresholding, and so on, which might have caused conflicting results. Moreover, fMRI studies are mostly based on tasks in different attention, inhibition, time, and motivation domain, and then, the results may not be precise enough. Future studies should minimize these differences and confounding variables to get a more convincing result.

## Conclusion

ADHD has far-ranging structural and functional abnormalities in the brain. The VBM meta-analysis found reduced GMV in the left superior frontal gyrus and the corpus callosum in patients with ADHD compared to HCs. Studies in task-related fMRI found that patients with ADHD showed low activation in the abovementioned brain regions. We hold the opinion that abnormal alterations in the structure and function of the left superior frontal gyrus and the corpus callosum may be the key brain regions involved in the pathogenesis of ADHD in patients and may be employed as an imaging metric for patients with ADHD pending future research. This meta-analysis also identified neuroanatomical or functional abnormalities in other brain regions in ADHD. Besides, subgroup analysis further explored and supported the above results, and this information can be applied to guide future studies. However, there is a need for more research in this area because of the lack of clarity on the confounding effects. Our results, as solid as it appears, should be read with caution.

## Data availability statement

The original contributions presented in the study are included in the article/supplementary material, further inquiries can be directed to the corresponding authors.

## Author contributions

MY and XG designed the experiment. MY, XG, XN, and MZ performed the experiment. ZY, SH, JC, and YZ modified the experiment and drafted the manuscript. All authors contributed to the article and provided approval for the final version of the manuscript.
